# The use of the white cell count and haemoglobin in combination as an effective screen to predict the normality of the full blood count

**DOI:** 10.1111/j.1751-553X.2011.01365.x

**Published:** 2012-02

**Authors:** A OSEI-BIMPONG, R McLEAN, E BHONDA, S M LEWIS

**Affiliations:** Imperial College Healthcare NHS Trust, Department of Haematology & WHO Collaboration Centre for Haematology TechnologyLondon, UK

**Keywords:** Haemoglobin, reference range, white cell count, screen, morphology

## Abstract

**Introduction:**

The utility of the full blood count (FBC) is vast with each parameter serving as a tool to aid diagnosis and monitor disease progression. However, the effectiveness of the test is hampered because of increased workload and lack of interpretation. In the effort to redress this issue, the combined use of the white blood cell count (WBC) and haemoglobin in predicting the normality of the FBC is evaluated.

**Method:**

FBC data were collated from 2191 patients and classified into two groups depending on whether the WBC and the haemoglobin were within the reference range. Blood films were examined on the abnormal FBC samples in each group and graded on morphology.

**Results:**

The FBC was normal in 89.6% of cases in the presence of a normal WBC and haemoglobin with subtle abnormalities in the remainder; 1+ grading of abnormal morphology in 93%. However, when the WBC and/or haemoglobin was abnormal, the remaining FBC was significantly abnormal (*P* < 0.05) and the corresponding blood films were grossly abnormal; 2+/3+ grading in 96% of cases.

**Conclusion:**

We concluded that in the presence of a normal WBC and haemoglobin, the FBC is normal in almost all cases and measuring these two parameters could be used as an effective screen to predict FBC normality.

## Introduction

The full blood count (FBC) is one of the most frequently requested pathological tests by clinicians and usually comprises 13–19 parameters with additional parameters continuously being introduced (Lewis, Bain & Bates, 2006).

The clinical utility of this test is vast; an increased or decreased total white blood cell count (WBC) could be due to abnormal bone marrow pathology (Leguit & van den Tweel, 2010). Leucocytosis with an associated neutrophilia or lymphocytosis could infer the presence of a microbial or viral infection (Estridge, Reynolds & Walters, 2000). A decrease in the WBC could also be caused by a chemotoxic effect during chemotherapy (Debled *et al.*, 2007). Clinicians can use the WBC biomarker to improve risk prognostication and identify patients in need of immediate treatment and a closer follow-up (Paladino *et al.*, 2010). A decrease in the red cell count and/or haemoglobin may infer anaemia, and depending on the red cell indices values, the aetiology of the anaemia can be deduced (Trent, 2006). The rate of increase in haemoglobin could be used to monitor the treatment of anaemia and determine the amount of blood required for transfusion (Taher, Musallam & Inati, 2009; Tettey *et al.*, 2009). The platelet count and its size can also be used to determine the thrombopoietic activity of the bone marrow, and an increase or decrease in platelet numbers can also point to disorders of haemostasis (Radaelli *et al.*, 2007) or liver disease (Adams, 2010).

The effectiveness of the FBC has been hampered because of a lack of appropriate screening, which in turn results in data overload (Miyakis *et al.*, 2006). A survey on clinicians and nurses revealed that nine of 10 end-users only utilized the WBC, haemoglobin and platelet of the 13 parameters reported routinely from the FBC profile because of either a lack of application or interpretation and time constraints (Pennycook, 1995).

Another reason for the underutilization of FBC data is the situation of large workload in most laboratories, such that those who are capable of interpreting all parameters within a FBC profile are inundated owing to the volume. As a result, decisions and follow-up actions on abnormal laboratory results are slowed down (Miyakis *et al.*, 2006).

Autovalidation criteria, which involve computerised algorithms to fast-track validation to transmit normal FBCs to the hospital information system, may reduce the workload on laboratory staff interpreting data and enable efficient procession of results; however, it would not resolve the issue of time and cost involved in analysing such large numbers of samples (Birkmeyer *et al.*, 2002). Therefore, there has been a rising demand to increase the diagnostic effectiveness of this test through appropriate screening (Pennycook, 1995; Miyakis *et al.*, 2006; Hawkins, 2007). With the advent of point-of-care testing, there are a few devices that measure single primary parameters such as WBC or haemoglobin accurately by a minute capillary (finger prick) sample (Morris, 2007; Osei-Bimpong *et al.*, 2009). Supporting such appropriate developments could represent opportunities for laboratory medicine to help influence the scope of future service delivery (Beastall, 2008). Testing for specific individual parameters on such devices is usually very quick with results obtained within minutes, and such testing is also low in cost (Morris, 2007; Osei-Bimpong *et al.*, 2009). The utility of an individual parameter such as the WBC or the haemoglobin, however, offers a limited and partial picture in the diagnosis (Kho *et al.*, 2007). Although it has been shown from a previous study that in majority of cases where the haemoglobin was within the normal range, the remainder of the FBC was normal (Lewis, Osei-Bimpong & Bradshaw, 2004), there are significant numbers where the WBC may be abnormal in the presence of a normal haemoglobin, notably in individuals with mild to moderate infections (Murphy *et al.*, 2007). Conversely, in the presence of a normal WBC, it is still possible to have a low haemoglobin and abnormal red cell indices, especially with most cases of nutritional anaemia (Subramanian, Kitson & Bhaniani, 2009). Thus, in such categories of patients, it is therefore not effective to screen or conclude comprehensively that the FBC is normal based on a single parameter such as the WBC or haemoglobin.

However, the utility of the WBC and haemoglobin in combination as a possible screen to predict the normality of the FBC has never been assessed; in this study, we determine the effectiveness of the use of the WBC in conjunction with the haemoglobin value to determine the outcome of the FBC; we determine the nature of the FBC profile when the WBC and the Haemoglobin are (i) both within the normal reference range and (ii) beyond the reference range. We compare the different extents of the FBC and blood film abnormalities in both groups.

## Methods

Over a 5-week period, FBC data were collated from a randomized selection of 2191 patients who had the test requested by their clinician in the Hammersmith Hospital Imperial College Healthcare Trust. The test served as an adjunct for the investigation of the underlying conditions.

This population included 42.6% women and 57.4% men, and the age range was 16–91 years. These comprised 39% of the patients referred from general practitioners, while 33% were hospitalized inpatients and the remainder were from outpatient departments.

### Sample collection

The samples were collected in 3-mL BD vacutainer® bottles (Becton Dickinson, Crowley, UK) containing 5.4 mg dipotassium EDTA anticoagulant. The samples were processed in their fresh state according to the standard laboratory protocol.

To control the nature of the counts more effectively, all samples were taken in the morning to control circadian rhythmic influences on the counts (Smaaland *et al.*, 2002).

### FBC analysis

The analyser on which the FBC samples were processed was the standard Sysmex XE2100 FBC analyser (Sysmex Corporation, Kobe, Japan); the analyser was adequately controlled by Internal Quality control that involved running an e-check® control once daily and also running a Levy–Jennings quality control programme to calculate the moving average for every 20 samples.

Access to all pathology test results, medical history and medication on each patient included in this study was obtained from the Laboratory and Hospital Informatics Systems.

### Reference range and screened groups

The reference range used for the FBC profile was established by locally deriving working reference values (Table 1) taking into account the wide age range of 16–91 years and the use a of a representative population (Lewis, Bain & Bates, 2006). The FBC data were then classified into two groups, i.e. normal screened group and abnormal screened groups depending on whether both the WBC and the haemoglobin were within or beyond the normal reference range.In each of the two groups, the number of samples that had the remaining FBC parameters within the reference range was recorded. Any sample that triggered an analyser flag was also classified as the abnormal group.

**Table 1:** Full blood count reference range

Parameters	Reference range	Reference range mean
White cell count (WBC)	4–11 × 10^9^/L	7.5 × 10^9^/L
Haemoglobin (Hb) (men)	13.0–17.0 g/dL	15.0 g/dL
Haemoglobin (women)	12.0–15.0 g/dL	13.5 g/dL
Platelets (Plt)	120–410 × 10^9^/L	275 × 10^9^/L
Neutrophils	2.0–8.0 × 10^9^/L	5.0 × 10^9^/L
Lymphocytes	1.0–3.5 × 10^9^/L	2.3 × 10^9^/L
Monocytes	0.1–1.0 × 10^9^/L	0.6 × 10^9^/L
Eosinophils	0.01–0.7 × 10^9^/L	0.4 × 10^9^/L
Basophils	0.01–0.1 × 10^9^/L	0.06 × 10^9^/L
Red cell count (RBC) (men)	3.80–5.50 × 10^12^/L	4.7 × 10^12^/L
RBC (women)	3.60–4.80 × 10^12^/L	4.2 × 10^12^/L
Mean cell volume (MCV)	83–101 fL	92 fL
Mean cell haemoglobin (MCH)	26–32 pg	29 pg
Mean cell haemoglobin concentration (MCHC)	31–35 pg	33 pg
Haematocrit (HCT) (men)	0.40–0.50 L/L	0.45 L/L
Haematocrit (women)	0.35–0.45 L/L	0.40 L/L

### Blood films

A corresponding blood film was prepared from all abnormal screened samples and examined microscopically in line with the standard laboratory procedure. The blood films were examined for morphological features and cell counts to verify the FBC and any associated analyser flags. The degree of abnormality was categorized using the ‘plus’ grading scale (HKTMA Haematology and Serology panel, 2002). The main morphological features and their associated grading criteria are shown in Table 2. Grading of the morphological abnormality was based on percentage or absolute numbers of cell subtypes observed in a 200-white cell differential under low-power magnification. (NCCLS, 1991).

**Table 2:** Morphological observation classification criteria

Morphological observation	1+ abnormal grading	2+ abnormal grading	3+ abnormal grading
Thrombocytosis (Plt × 10^9^/L)	411–500	501–650	>650
Thrombocytopenia (Plt × 10^9^/L)	100–119	50–99	<50
Granulocytosis (WBC × 10^9^/L)	9–17	18–26	>26
Granulocytopenia (WBC × 10^9^/L)	0.5–1.0	0.25–0.49	<0.25
Lymphopenia (WBC × 10^9^/L)	0.5–1.0	0.25–0.49	<0.25
Lymphocytosis/variant forms	>6%	>12%	>18%
Monocytosis	>7%	>14%	>21%
Eosinophilia	>7%	>14%	>21%
Immature leucocytes/left shift	>7%	>14%	>21%
Nucleated red cells	>2%	>4%	>6%
Hypochromia	2–4%	5–7%	8–20%
Microcytosis	5–20%	21–50%	51–75%
Macrocytosis	2–10%	11–25%	26–50%
Polychromasia	3–5%	5–25%	>25%
Red cell inclusions	<1%	2–3%	>3%
Spherocytosis	1–5%	5–25%	>25%

### Statistical Methods

All data were analysed using Excel software statistics package analysis software (Microsoft Office Excel® 2003); in each of the two groups, the range and the standard deviation were calculated.

Student’s paired *t*-tests were also calculated using this package; *P-*values <0.05 were considered significant.

## Results

A total of 939 FBC profiles were within the normal screened group where both the WBC and haemoglobin (Hb) values fell within the normal reference range: WBC = 4–11 × 10^9^/L and Hb 13–17 g/dL (men), Hb 12–15 g/dL (women). In this group, there were 841 (89.6%) samples in which the remainder of the FBC was also within the normal reference range, i.e. there were only 98 cases in which there was an abnormal parameter/analyser flag for the remaining FBC.

In the abnormal screened group where either or both WBC and haemoglobin fell outside the reference limits, there were 1252 FBC profiles in this group, and in all cases, there was an additional parameter with a value beyond the reference limit, i.e. none of the samples in this latter category had a normal value for the remaining FBC parameters.

In both groups, the numbers of samples were classified detailing the numbers of cases outside the limits for each of the FBC parameters (Tables 3 and 4). A morphology comment was made on the corresponding blood film from each sample. (Table 5) The degree of abnormality was also evaluated and verified by examining the corresponding blood films of the samples with abnormal FBC values; these were further classified/graded depending on the degree of abnormality.

**Table 3:** Percentage number of samples outside reference limits and data range for white cell differential in two screened groups

	Neutrophils	Lymphocytes	Monocytes	Eosinophils	Basophils
Normal screened group					
% No. of samples outside limits of WBC subtype [data range (×10^9^/L)/standard deviation]	1.9 (1.1–9.5/1.29)	4.9 (0.3–5.1/0.71)	5.8 (0.1–1.4/0.16)	4.0 (90.0–1.60/0.22)	0.1 (0.0–0.14/0.017)
Abnormal screened group					
% No. of samples outside reference limits of WBC subtype [data range (×10^9^/L)/standard deviation]	36.9 (0.21–37.74/5.97)	27.0 (0.1–12.2/1.38)	37.0 (0.1–2.94/0.436)	17.0 (0.1–4.92/0.32)	3.2 (0.0–2.49/0.11)
*P* value	0.0082	0.0072	0.0010	0.0026	0.0029

**Table 4:** Percentage number of samples outside reference limits and data range for remaining full blood count (red cells and platelets) in two screened groups

	Plts (×10^9^/L)	RBC (×10^12^/L)	HCT (L/L)	MCV (fL)	MCH (pg)	MCHC (g/L)
Normal screened group						
% No. of samples outside reference limits (data range/standard deviation)	2.2 (93–477/67.1)	0.6 (3.80–5.82/0.38)	0.6 (0.336–0.517/0.262)	2.1 (76.9–105.8/4.9)	4.9 (24.6–35.7/0.33)	3.1 (29.3–37.8/0.99)
Abnormal screened group						
% No. of samples outside reference limits (data range/standard deviation)	29.1 (8–812/123))	39.0 (1.44–7.34/0.87)	39.0 (0.14–0.593/0.619)	21.5 (57.6–118.4/7.61)	25.0 (18.8–43.7/2.79)	32.0 (26.1–43.6/1.43)
*P* value	0.0006	0.0030	0.0029	0.0006	0.0067	0.0074

Plts, platelets; RBC, red blood count; MCV, mean cell volume; MCH, mean cell haemoglobin; MCHC, mean cell haemoglobin concentration; HCT, haematocrit.

**Table 5:** Morphology grading distribution on blood film for samples with analyser flag/full blood count (FBC) values beyond reference limits

	% No. of samples with normal film	% No. of samples with abnormal film of 1+ grading	% No. of samples with abnormal film of 2+ grading	% No. of samples with abnormal film of 3+ grading
Normal screened group failing reference limits for remaining FBC parameters (*N* = 98)	7	93	0	0
Abnormal screened group failing reference limits for remaining FBC parameters (*N* = 1252)	0	4	64	32

## Discussion

All white cell, red cell and platelet parameter ranges were significantly closer to the corresponding reference range in the normal screened group in comparison with the abnormal screened group: Within the normal screened group, the WBC differential was normal in most cases, and the data range for each of the white cell subtypes was significantly narrower in comparison with the abnormal screened group (Table 3), *(P* < 0.05*)*. We observed that there were considerably few patients in the normal screened group (1.9% beyond the reference limit for neutrophils) and the deviation was subtle and clinically insignificant; all their corresponding blood films reflected normal morphology (Table 5). It has been shown that these slightly abnormal counts are usually caused by physiological changes associated with certain conditions such as pregnancy whereby the count returns to normal after delivery (Dale & Liles, 2003), and also abnormally low neutrophil counts are found in approximately 25–50% of persons of African descent who have benign ethnic neutropenia without evidence of increased susceptibility to infection or any other adverse effects (Haddy, Rana & Castro, 1999).

The red cells and their indices were also within the normal reference limits in most cases when the WBC and the haemoglobin were normal (Table 4). The red cell count was beyond the reference limits in only 0.6% of cases in comparison with the abnormal screened group where 39% of patients had an abnormal red cell count. The degree to which they were abnormal was also significantly wider (*P* < 0.05), and the SD values were higher in all red cell parameters. On examining the corresponding blood films of samples beyond the reference range within the normal screened category, none of the morphological features were classified as >1+ grading (Table 5). The haematinics were also checked; B12, serum folate and the iron status in these patients were all found to be within the normal ranges, and in these patients, the marginally low red cell indices may be attributed to heterozygous cases of haemoglobinopathies, such as alpha thalassaemia minor, most of which are asymptomatic (Hattori, 2002).

The platelet count was outside the normal reference range in only 2.2% of samples with a normal WBC and haemoglobin. However, when either or both the WBC and haemoglobin were abnormal, a normal platelet count was beyond the reference range in 29.1% of cases and the degree to which the value deviated from the normal range was significantly higher (platelet count range: 8–812 × 10^9^/L, *P* < 0.05) in comparison with the cases where both the WBC and the haemoglobin were normal (platelet count range: 93–477 × 10^9^/L).

Thus, we have shown such an effective screen that measures WBC and haemoglobin to assure laboratory and/or clinical staff of the high probability of FBC normality. This could streamline the numbers to enable laboratory staff to focus on interpreting abnormal FBCs to optimize patient care more speedily and effectively; testing can be streamlined to identify and focus on more likely abnormal FBC and blood films. In cases where the WBC and haemoglobin are normal, resources could be then channelled into carrying out other pathological tests instead to arrive at a correct diagnosis. In that way, maximum information would be obtained from laboratory tests to provide greatest benefit at least cost.

Another advantage of measuring only WBC and haemoglobin is the speed, with a WBC achieved in minutes using the Hemocue WBC® (Osei-Bimpong *et al.*, 2009) and the haemoglobin obtainable within a minute using the Hemocue Hb 301® (HemoCue AB, Angelholm, Sweden); the key advantage of such point-of-care testing is the rapid time from sampling to the result being involved in a clinical decision (Morris, 2007).

Occasionally, there are difficulties in obtaining adequate sample for an FBC in some patients because of collapsed veins, and most of these cases, the clinician may just need to ascertain whether there has been an adequate increment in the haemoglobin, post-transfusion or whether there has been an adequate response to antibiotics reflected in the resolve of a leucocytosis; in such cases, a finger prick to determine the WBC or haemoglobin would suffice, and with the advent of devices, within which these parameters can be determined accurately, there would be a high utility.

In developing countries, and in many rural primary health centres, this may be the more cost-effective approach compared with the running of a FBC, making savings on the sample tubes and the FBC analyser reagents. A further benefit could be to reduce the travel of patient to a location where FBC testing is performed. Point-of-care testing of the WBC and haemoglobin may offer greater area coverage especially in the rural or under-resourced population where fewer laboratories may be available (Bates & Mendelow, 2006). It could also be of value, especially in general practice and in rural clinics without access to FBC analysers.

There are also numerous circumstances where adequate facilities may not be readily available for full profile diagnostic tests such as the FBC, especially in disaster areas and war zones (Kost *et al.*, 2006), and a speedy diagnostic technique may be required; the determination of the WBC and the haemoglobin could be an effective screen to triage any potentially abnormal FBCs to warrant further haematological investigations.

## Conclusion

We have shown that in a wide and largely randomized selection of FBCs, in majority of cases where the WBC and the haemoglobin are within the normal range, the remainder of the FBC profile is normal as well.

In a minority of cases, where the WBC and the haemoglobin are normal, the FBC profile could have one or more parameters outside the reference range; however, the degree is remarkably subtle and of little clinical significance.
